# Increased FAT/CD36 Cycling and Lipid Accumulation in Myotubes Derived from Obese Type 2 Diabetic Patients

**DOI:** 10.1371/journal.pone.0028981

**Published:** 2011-12-16

**Authors:** Celine Aguer, Marc Foretz, Louise Lantier, Sophie Hebrard, Benoit Viollet, Jacques Mercier, Magali Kitzmann

**Affiliations:** 1 INSERM, U1046 Physiologie et Médecine Expérimentale du Cœur et des Muscles, Montpellier, France; 2 Université Montpellier 1, Université Montpellier 2, Montpellier, France; 3 Inserm, U1016, Institut Cochin, Paris, France; 4 CNRS, UMR8104, Paris, France; 5 Université Paris Descartes, Paris, France; 6 CHRU Montpellier, Montpellier, France; Clermont Université, France

## Abstract

**Background:**

Permanent fatty acid translocase (FAT/)CD36 relocation has previously been shown to be related to abnormal lipid accumulation in the skeletal muscle of type 2 diabetic patients, however mechanisms responsible for the regulation of FAT/CD36 expression and localization are not well characterized in human skeletal muscle.

**Methodology/Principal Findings:**

Primary muscle cells derived from obese type 2 diabetic patients (OBT2D) and from healthy subjects (Control) were used to examine the regulation of FAT/CD36. We showed that compared to Control myotubes, FAT/CD36 was continuously cycling between intracellular compartments and the cell surface in OBT2D myotubes, independently of lipid raft association, leading to increased cell surface FAT/CD36 localization and lipid accumulation. Moreover, we showed that FAT/CD36 cycling and lipid accumulation were specific to myotubes and were not observed in reserve cells. However, in Control myotubes, the induction of FAT/CD36 membrane translocation by the activation of (AMP)-activated protein kinase (AMPK) pathway did not increase lipid accumulation. This result can be explained by the fact that pharmacological activation of AMPK leads to increased mitochondrial beta-oxidation in Control cells.

**Conclusion/Significance:**

Lipid accumulation in myotubes derived from obese type 2 diabetic patients arises from abnormal FAT/CD36 cycling while lipid accumulation in Control cells results from an equilibrium between lipid uptake and oxidation. As such, inhibiting FAT/CD36 cycling in the skeletal muscle of obese type 2 diabetic patients should be sufficient to diminish lipid accumulation.

## Introduction

Intramyocellular lipid accumulation in skeletal muscle is abnormally high in type 2 diabetes and contributes to the etiology of the pathology [1]. Long chain fatty acid (such as palmitate) uptake is achieved by a concert of co-existing mechanisms. These lipids can passively diffuse, but certain membrane proteins can also accelerate the transport. Membrane fatty acid transporters can modulate the balance between fatty acid uptake and subsequent storage and/or oxidation in muscle tissue. The principal muscle fatty acid transporter FAT/CD36 (fatty acid translocase) is involved in regulating the uptake of long-chain fatty acids into skeletal muscle [2], [3]. Abnormal increased fatty acid transport [4] and membrane FAT/CD36 relocation [4], [5] independent of mitochondrial dysfunction result in an excessive accumulation of intramyocellular lipid in skeletal muscle tissue of type 2 diabetic patients. To date, little is known about FAT/CD36 regulation in human skeletal muscle. In this tissue, the mechanisms known to induce FAT/CD36 translocation from intracellular storage compartments towards plasma membrane are insulin, (AMP)-activated protein kinase (AMPK) signaling pathways and muscle contraction [6], [7].

Mature muscles possess a population of satellite cells located on their surface. *In vitro* satellite cells differentiation is characterized by withdrawal of myoblasts from the cell cycle, induction of muscle-specific gene expression, and cell fusion into multinucleated myotubes. After differentiation *in vitro*, satellite cells are composed of two populations of cells: mature myotubes and quiescent undifferentiated myoblasts (reserve cells) [8]. In adipocytes, FAT/CD36 expression appeared to be closely linked to preadipocyte differentiation [9], however, the regulation of FAT/CD36 expression and localization during the differentiation of primary human satellite cells has not been previously studied. These primary human satellite cells, when derived from type 2 diabetic patients, have been shown to display the majority of the defects previously described for type 2 diabetic muscle in vivo including defective insulin signaling pathway [10], [11], [12], metabolic inflexibility [13], [14], [15] and abnormal cell surface FAT/CD36 localization [5].

In this context, cell culture of human primary satellite cells offers an excellent and dynamic model to study the link between lipid accumulation and the regulation of FAT/CD36 localization. The aim of this study was to compare FAT/CD36 regulation between primary human satellite cells isolated from healthy subjects and from obese type 2 diabetic patients [5] in order to uncover new mechanisms of FAT/CD36 regulation associated with obesity and/or type 2 diabetes.

## Results

### Plasma membrane FAT/CD36 and lipid accumulation are increased in OBT2D cells

**Figure 1:**
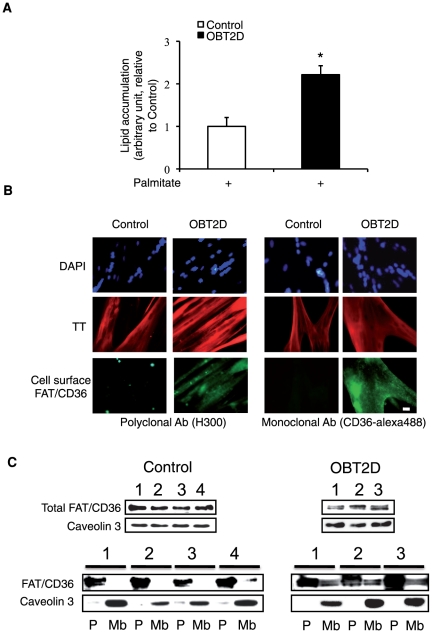
OBT2D myotubes are characterized by increased lipid accumulation and plasma membrane FAT/CD36 localization. A. Quantification of lipid accumulation in Control (n = 4) and OBT2D differentiated satellite cells (n = 5) after palmitate treatment (0.6 mM for 16 h). Data are presented normalized to lipid accumulation in Control myotubes. Each point was assayed in triplicate for each of the 9 independent cell cultures. *, p<0.05, OBT2D versus Control differentiated cells. B. Representative immunofluorescence microscopy of satellite cells established from control subjects (Control) and obese type 2 diabetic patients (OBT2D) after 8 days of differentiation. Living myotubes were incubated for 1 h with an antibody against FAT/CD36 (H300, left panel) and for 1 h with an antibody against FAT/CD36 alexa 488 (CD36- alexa488, right panel). For H300 staining a polyclonal secondary antibody conjugated to alexa 488 (green) was added to the cells for 1 h. After fixation and permeabilization, cells were incubated with an antibody against troponin T (TT) visualized using a secondary monoclonal antibody conjugated to alexa 546 (red). Nuclei in cells were stained by dapi (blue). The 4 Control and the 5 OBT2D showed a staining similar to the representative pictures. Scale bar represents 30 µm. C. Top panels: Western blot analysis of total FAT/CD36 and caveolin 3 expression in differentiated satellite cell lysate established from 4 control subjects numbered 1 to 4 (Control) and 3 obese type 2 diabetic (OBT2D) patients numbered 1 to 3. Bottom panels: Western blot analysis of FAT/CD36 and caveolin 3 expression in post-membrane fractions (P) and plasma membrane fractions (Mb) of differentiated satellite cells established from 4 control subjects (1 to 4) and 3 obese type 2 diabetic (1 to 3) patients.

We have previously shown that lipid accumulation was dependent upon the obesity of the donor subject and partially due to cell surface FAT/CD36 localization [5]. We confirmed this result by showing that palmitate-induced lipid accumulation was significantly increased in myotubes derived from obese type 2 diabetic patients (OBT2D) compared to myotubes derived from non-obese healthy subjects (Control) (p<0.05; Fig. 1 A). We also confirmed cell surface localization of FAT/CD36 in OBT2D cells, by immunofluorescence staining of FAT/CD36 in living cells (no permeabilization) using two different antibodies raised against FAT/CD36, a polyclonal antibody (H300) (Fig. 1 B, left panel) and a monoclonal conjugated to alexa488 antibody (CD36-alexa488) (Fig. 1 B, right panel). Moreover, cell surface localization of FAT/CD36 was specifically observed in multinucleated troponin T (TT) positive OBT2D myotubes with both antibodies (left and right panels), with no such staining in Control cells. In order to show that cell surface staining of FAT/CD36 was quantitatively associated with plasma membrane relocation, we performed fractionation experiments and prepared plasma membrane (Mb) and post-plasma membrane (P) fractions (Fig. 1 C). Since caveolin 3 is expressed exclusively in muscle cell types [16] and is expressed in plasma membrane microdomains, it was used as a marker of plasma membrane fractions. We monitored total FAT/CD36 and caveolin 3 expression in Control derived from four different healthy subjects (1 to 4) and in OBT2D derived from three obese type 2 diabetic patients (1 to 3) cells before fractionation (Fig. 1 C, top panels). After fractionation (Fig. 1 C, bottom panels), FAT/CD36 in Control (1 to 4) differentiated cells was only detected in caveolin 3 negative P fractions. In contrast, in OBT2D (1 to 3) differentiated cells, FAT/CD36 was found in both P and Mb fractions.

### FAT/CD36 membrane localization in OBT2D myotubes is involved in lipid accumulation

**Figure 2:**
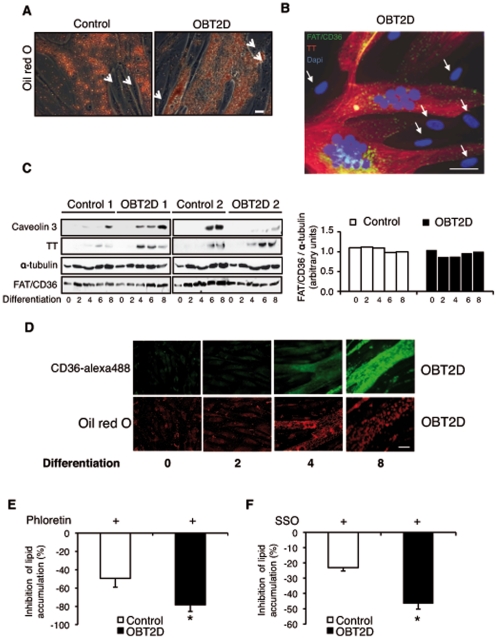
Increased membrane localization of FAT/CD36 during differentiation is responsible for increased lipid accumulation in OBT2D myotubes. A. Representative light microscopy of myotubes derived from control subjects (Control) or obese type 2 diabetic patients (OBT2D), after 8 days of differentiation, stained by oil red O after palmitate treatment (0.6 mM for 16 h). The 4 Control and the 5 OBT2D cells showed a staining similar to the representative pictures. Arrows show reserve cells. Scale bar represents 30 µm. B. Merged picture of FAT/CD36 (H300), troponin T (TT) and dapi staining in OBT2D differentiated cells (for 8 days). Living cells were incubated for 1 h with an antibody against FAT/CD36 (H300, left panel) and for 1 h with a polyclonal secondary antibody conjugated to alexa 488 (green). After fixation and permeabilization, cells were incubated with an antibody against troponin T (TT) visualized using a secondary monoclonal antibody conjugated to alexa 546 (red). The 5 OBT2D cells showed a staining similar to the representative pictures. Arrows show reserve cells. Scale bar represents 30 µm. C. Left panel: Western blot analysis of the expression of total FAT/CD36 in proliferative (0), and after 2, 4, 6 and 8 days of differentiation of cells established from two control subjects (Control 1 and 2) and two obese type 2 diabetic patients (OBT2D 1 and 2). Troponin T (TT) and caveolin 3 were used as markers of myotube differentiation and α-tubulin as a loading control. Right panel: quantification by density analysis of the 2 controls and the 2 OBT2D. Data are presented normalized to α-tubulin protein expression where the value of Control cells after 8 days of differentiation has been arbitrary chosen as the reference value equal to 1. D. Representative immunofluorescence microscopy of cells established from obese type 2 diabetic patients (OBT2D) in proliferative (0), and after 2, 4 and 8 days of differentiation, treated by palmitate (0.6 mM for 16 h), incubated for the last hour with CD36-alexa488 antibody (green) and stained by oil red O (red) after fixation. The 5 OBT2D cells showed a staining similar to the representative pictures. Scale bar represents 30 µm. E. Percentage of inhibition of lipid content in Control (n = 4) and in OBT2D differentiated satellite cells (n = 5) after phloretin stimulation (400 µM for 30 min) followed by palmitate treatment (0.6 mM for 16 h). Data are means ±SEM. Each point was assayed in triplicate for each of the 9 independent cell cultures. *, p<0.05, OBT2D versus Control cells. F. Percentage of inhibition of lipid content in Control (n = 4) and OBT2D differentiated satellite cells (n = 4) after SSO stimulation (250 µg/ml for 30 min) followed by three PBS washes and by palmitate treatment (0.6 mM for 16 h). Data are means ±SEM. Each point was assayed in triplicate for each of the 8 independent cell cultures. *, p<0.05, OBT2D versus Control cells.

During skeletal muscle differentiation, myoblasts exit the cell cycle and differentiate, giving rise to a heterogeneous population of cells. A major subpopulation consists of myotubes, quiescent multinucleated cells expressing muscle-specific differentiation proteins such as troponin T. A minor subpopulation of myoblasts remains quiescent and undifferentiated [8]. Positive oil red O staining was only observed in multinucleated Control and OBT2D myotubes but not in reserve cells (Fig. 2 A, arrows show reserve cells). As shown in figure 2 B (overlay of FAT/CD36, troponin T and DAPI staining) cell surface FAT/CD36 appeared as a punctuate staining only detected at the cell surface of troponin T positive cells and not in reserve cells (arrows). To determine if the specific localization of FAT/CD36 in OBT2D myotubes was due to increased expression of FAT/CD36 during differentiation, we monitored total FAT/CD36 expression by Western blot in two Control (1 and 2) and two OBT2D (1 and 2) cell cultures (0, 2, 4, 6 and 8 days of differentiation) (Fig. 2 C). α-tubulin expression was used as a loading control. OBT2D satellite cells have a normal induction of troponin T and caveolin 3 expression during differentiation. Total FAT/CD36 expression was already detected in proliferative cells and was not increased during differentiation in Control and OBT2D cells (Fig. 2 C, right panel).

Plasma membrane FAT/CD36 and lipid accumulation were observed in differentiated OBT2D cells. To determine whether a positive correlation between membrane FAT/CD36 and lipid accumulation could be observed in OBT2D cells, cell surface FAT/CD36 and oil red O co-staining was performed during differentiation. Positive oil red O and membrane FAT/CD36 co-staining was only detected in differentiated cells, after 4 and 8 days of differentiation (Fig. 2 D). We next determine whether FAT/CD36 membrane localization and lipid accumulation were linked in OBT2D myotubes. Palmitate uptake occurs through passive diffusion and protein-mediated transport. Phloretin is a general transport inhibitor [17]. We tested the possible influence of phloretin (Fig. 2 E) prior to palmitate treatment. Phloretin inhibited palmitate-induced lipid accumulation to a greater extend in OBT2D cells than in control cells (−78.4±7.3% versus −49.4±9.5%, *p*<0.05). We next tested the possible influence of sulfo-N-succinimidyloleate (SSO), which specifically binds to plasma membrane FAT/CD36, resulting in an arrest of the transport function of this protein [18] (Fig. 2 F). Incubation of Control and OBT2D differentiated cells with SSO inhibited palmitate-induced lipid accumulation. Again, the inhibition was significantly more pronounced in OBT2D cells than in Control cells (−46.6±3.7% versus −23.0±2.2%, *p*<0.05).

### Increased translocation of FAT/CD36 in Control cells after AMPK activation or insulin stimulation

**Figure 3:**
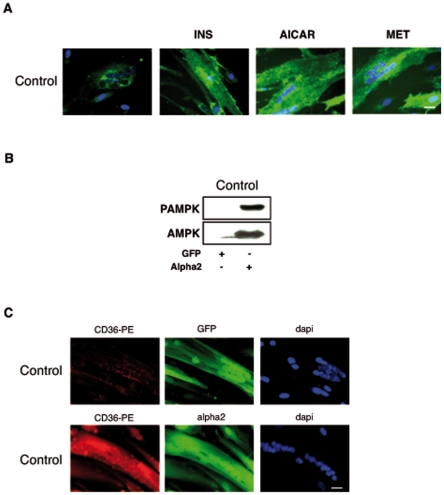
AMPK activation increases FAT/CD36 translocation in Control myotubes. A. Representative immunofluorescence microscopy of myotubes established from control subjects (Control) after 8 days of differentiation, incubated for 1 h at 37°C with CD36-alexa488 antibody (green) followed by insulin stimulation (100 nM for 10 min) or by AICAR stimulation (500 µM for 1 h) or by metformin stimulation (2 mM for 1 h). Nuclei were stained by dapi (blue) after fixation of the cells. The 4 Control cells showed a staining similar to the representative pictures. Scale bar represents 30 µm. B. Western blot analysis of the expression of AMPK and the phosphorylated form of AMPK (PAMPK) after infection with an adenovirus expressing either GFP (GFP) or GFP and the constitutively activated form of AMPK alpha 2 (alpha 2) in differentiated cells established from control subjects (Control). C. Representative immunofluorescence microscopy of myotubes established from control subjects (Control) after 8 days of differentiation infected with an adenovirus expressing either GFP (GFP) or GFP and the constitutively activated form of AMPK alpha 2 (alpha 2). To monitor cell surface FAT/CD36 localization in a co-staining experiment, CD36-alexa488 antibody (green) could not be used because of the GFP expression, as such, the same antibody against FAT/CD36 was used but with PhytoErythrine (CD36-PE) as a red fluorochrome. Living cells were incubated for 1 h with CD36- PE (red). Nuclei in cells were stained by dapi (blue). Scale bar represents 30 µm.

To test whether translocation of FAT/CD36 in Control myotubes could be increased, cells were acutely treated with AMPK activators (AICAR and metformin (MET)) or insulin (INS), two stimuli known to induce the translocation of FAT/CD36 to the plasma membrane of muscle tissue [7] (Fig. 3 A). Acute INS, AICAR or MET treatments induced FAT/CD36 cell surface localization in Control myotubes. We also overexpressed a constitutively activated form of AMPKalpha 2 subunit [19] in Control cells. This constitutively activated form of AMPK alpha2 was well expressed and phosphorylated (Fig. 3 B). FAT/CD36 translocation (in red) in Control cells was observed after AMPK alpha2 overexpression (Fig. 3 C) whereas FAT/CD36 was not localized to the plasma membrane after infection with the empty adenovirus (GFP).

### AMPK activation by AICAR induced increased beta-oxidation and no change in lipid accumulation in Control cells

**Figure 4:**
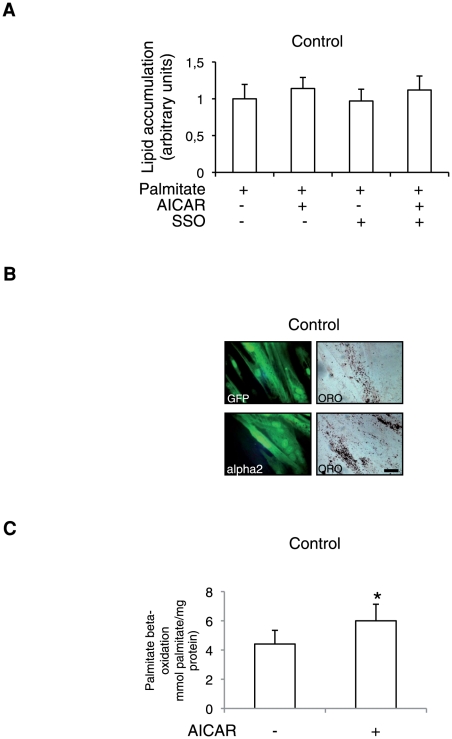
AMPK-mediated FAT/CD36 translocation in Control cells does not modify lipid accumulation. A. Quantification of lipid accumulation in Control (n = 4) (A) after palmitate treatment (0.6 mM for 16 h) or palmitate treatment plus AICAR stimulation (500 µM for 1 h) or palmitate treatment after SSO addition (250 µg/ml for 30 min) or palmitate treatment with or without AICAR stimulation after SSO addition. Data are means ±SEM. Each point was assayed in triplicate for each of the 4 independent cell cultures. B. Representative fluorescence and light microscopy of myotubes established from control subjects (Control) and from obese type 2 diabetic patients (OBT2D) after 8 days of differentiation infected with an adenovirus expressing either GFP (GFP) or GFP and the constitutively activated form of AMPK alpha 2 (alpha 2) stained by oil red O (ORO) after palmitate treatment (0.6 mM for 16 h). C. Palmitate beta-oxidation in differentiated Control cells (n = 3) before (−) and after (+) AICAR stimulation (500 µM for 1 h) expressed relative to protein content. Experiments were performed in triplicate for each of the 3 independent cell cultures. Data are mean ±SEM. *, P<0.05, AICAR-treated versus untreated Control cells.

To determine whether increased translocation of FAT/CD36 in Control myotubes was associated with increased lipid accumulation, we tested the effect of acute AICAR stimulation alone or in combination with specific FAT/CD36 inhibition (SSO) on lipid accumulation. SSO treatment did not significantly reduce lipid accumulation in Control cells and AICAR treatment did not induce increased lipid accumulation (Fig. 4 A). We also tested the effect of expressing the constitutively activated form of AMPK alpha 2 on lipid accumulation (Fig. 4 B) and found that Control myotubes overexpressing AMPK alpha 2 did not show increased positive oil red O cells. Lipid accumulation in myotubes is the result of the balance between FA uptake, FA esterification and FA oxidation. Since AMPK activation is known to induce increased mitochondrial beta oxidation [20] we measured beta oxidation before and after AICAR stimulation in Control cells. As shown on figure 4 C, beta-oxidation was significantly increased by acute AICAR treatment (p<0.05).

### FAT/CD36 is not associated with lipid rafts in OBT2D myotubes

**Figure 5:**
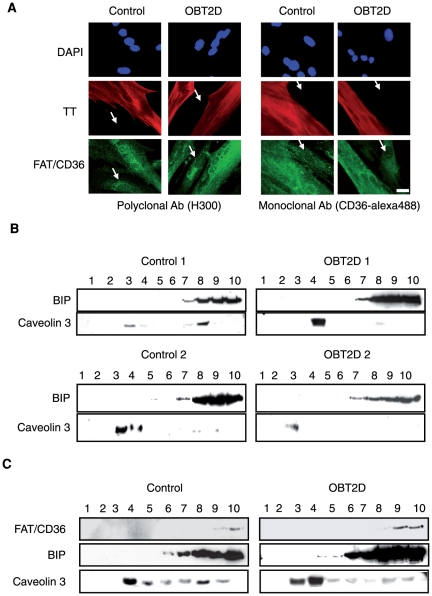
Plasma membrane FAT/CD36 is not associated to lipid raft in OBT2D myotubes. A. Representative immunofluorescence microscopy of satellite cells established from control subjects (Control) and obese type 2 diabetic patients (OBT2D) after 8 days of differentiation, incubated after fixation and permeabilization with antibodies against FAT/CD36 (H300, left panel and CD36- alexa488, right panel) and against troponin T (TT). A polyclonal secondary antibody conjugated to alexa 488 (green) was used to visualize H300 and a secondary monoclonal antibody conjugated to alexa 546 (red) to visualize troponin T. Nuclei in cells were stained by dapi (blue). The 4 Control and the 5 OBT2D cells showed a staining similar to the representative pictures. Arrows show reserve cells. Scale bar represents 30 µm. B. Differentiated satellite cells derived from two control subjects (Control 1 and 2) (left panels) and from two obese type 2 diabetic patients (OBT2D 1 and 2) (right panels) were lysed in 1% Triton and subjected to flotation sucrose density gradient centrifugation. Equal volumes of each fraction (1 to 10) were analyzed by western blotting with antibodies against BIP and caveolin 3. Caveolin 3 mark detergent resistant membranes (lipid rafts) fractions. Non-raft proteins resident in the endoplasmic reticulum (BIP) are recovered in heavier fractions. C. Same experiment as in (B) using BIP, caveolin 3 and FAT/CD36 (H300) antibodies. The 4 Control and the 5 OBT2D cell cultures showed a FAT/CD36 expression similar to the representative Western blots.

In order to gain insight into mechanisms of FAT/CD36 localization in OBT2D myotubes, we examined intracellular staining of FAT/CD36 (after fixation and permeabilization) in Control and OBT2D muscle cells. Intracellular FAT/CD36 appeared similar between Control and OBT2D cells when using the two antibodies directed against FAT/CD36, H300 (Fig. 5 A, left panel) and CD36-alexa488 (Fig. 5 A, right panel). Moreover, intracellular FAT/CD36 was observed in both differentiated cells (troponin T positive) and reserve cells (arrows). In adipocytes, FAT/CD36 has been found in lipid rafts and caveolae [21], suggesting that lipid rafts regulate the expression and function of FAT/CD36 at the level of the plasma membrane. Lipid rafts are composed of DRMs (detergent resistant-membranes). Isolation of DRMs was performed in order to compare lipid rafts between Control and OBT2D differentiated cells. Caveolin 3 expression was used to monitor DRMs purification and the molecular chaperone binding protein (BIP) as an endoplasmic reticulum marker excluded from DRMs. As shown on Figure 5 B, caveolin 3 expression was variable between cells (Control 1, Control 2, OBT2D 1 and OBT2D 2) but was mainly found in fractions 3 and/or 4 (identified as DRMs) in Control and in OBT2D differentiated cells. Extraction with Triton X-100 yielded good separation of lipid rafts from BIP, as it was not detected in DRM fractions. The association of FAT/CD36 with lipid rafts was then analyzed. Surprisingly, FAT/CD36 expression was only detected in fractions 9 and 10 in both Control and OBT2D cells (Fig. 5 C) but not in DRMs fractions showing that FAT/CD36 is not associated with lipid rafts in primary human myotubes.

### Increased FAT/CD36 cycling in OBT2D myotubes

**Figure 6:**
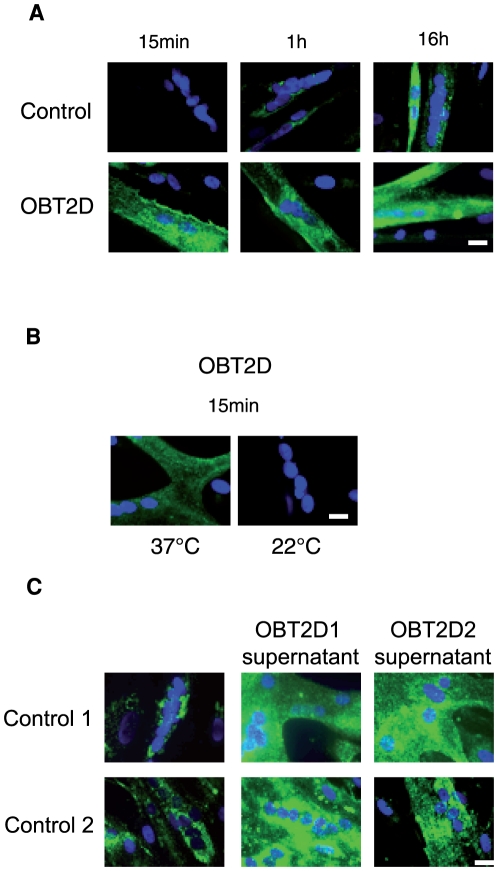
FAT/CD36 is continuously cycling in OBT2D myotubes. A. Representative immunofluorescence microscopy of myotubes established from control subjects (Control) (top panels) and from obese type 2 diabetic patients (OBT2D) (bottom panels) after 8 days of differentiation, incubated on living cells for 15 min, 1 h and 16 h at 37°C with an antibody against FAT/CD36 alexa 488 (CD36- alexa488). Nuclei in cells were stained by dapi (blue). The 4 Control and the 5 OBT2D showed a staining similar to the representative picture. Scale bar represents 30 µm. B. Representative immunofluorescence microscopy of myotubes established from obese type 2 diabetic patients (OBT2D) after 8 days of differentiation, incubated on living cells for 15 min at 37°C and at 22°C with an antibody against FAT/CD36 alexa 488 (CD36- alexa488). Nuclei in cells were stained by dapi (blue). The 5 OBT2D cells showed a staining similar to the representative pictures. Scale bar represents 30 µm. C. Representative immunofluorescence microscopy of myotubes established from control subjects (Control 1 and Control 2) after 7 days of differentiation, incubated overnight with supernatant from OBT2D1 or OBT2D2 and for 1 h at 37°C with an antibody against FAT/CD36 alexa 488 (CD36- alexa488). Nuclei in cells were stained by dapi (blue). Scale bar represents 30 µm.

In order to monitor the dynamic of FAT/CD36 cycling, we performed immunofluorescence staining of FAT/CD36 in living cells by incubating CD36-alexa488 antibody for different periods of time (15 min, 1 h and 16 h). Incubation of living Control cells with CD36-alexa 488 antibody only showed important cell surface FAT/CD36 staining after 16 h (Fig. 6 A, top panels). Conversely, cell surface FAT/CD36 was already detected in OBT2D myotubes after 15 min of incubation and was independent of incubation time (Fig. 6 A, bottom panels). We incubated CD36-alexa488 at a lower temperature (room temperature, 22°C) to reduce the rate of FAT/CD36 cycling in OBT2D myotubes. As shown on Figure 6 B, cell surface FAT/CD36 was no longer detected in OBT2D myotubes after 15 min of incubation with the antibody at 22° C. To test the hypothesis that factors released by OBT2D myotubes in the medium were responsible for FAT/CD36 cycling, we tested supernatant of OBT2D differentiated cells on Control differentiated cells (Fig. 6 C). Interestingly, supernatants from two different OBT2D differentiated cells (OBT2D1 and OBT2D2) increased cell surface FAT/CD36 in two different Control differentiated cells (Control 1 and Control 2).

## Discussion

### Primary human muscle cells represent a good model for studying molecular mechanisms involved in type 2 diabetes

Primary human satellite cells have been shown to display the majority of the defects previously described for type 2 diabetic muscle in vivo including defective insulin signaling pathway [10], [11], reduced insulin-stimulated glycogen synthase and glucose transport activities [22], [23], and reduced lipid oxidation [24], [25]. We have previously shown an abnormal cell surface FAT/CD36 localization [5] in cells derived from obese patients and an abnormal metabolic flexibility in response to high palmitate concentrations in myotubes derived from obese type 2 diabetic patients [15]. In the present study we have obtained new interesting results, which confirm that this cellular model can be used to study mechanisms related to type 2 diabetes in human skeletal muscle. By performing fractionation experiments, we were able to show biochemically that using CD36-alexa 488 antibody in living cells is a simple and powerful tool to follow cell surface localization of FAT/CD36. Moreover, this tool can be used to test molecules involved in FAT/CD36 translocation since we have shown that signalling pathways (insulin and AMPK) involved in FAT/CD36 translocation in the skeletal muscle [6], [7] were also able to induce endogenous FAT/CD36 translocation in Control cells. A permanent relocation of FAT/CD36 was observed in the skeletal muscle tissue of obese and T2D patients [4], [5]. The term permanent relocation was used since the increase in membrane-associated FAT/CD36 [4], [26] was accompanied by a decrease in intracellular (vesicle-bound) FAT/CD36 and not by an increase in total FAT/CD36 expression. In our study, we have shown that total FAT/CD36 expression was not increased in OBT2D cells. Cell surface FAT/CD36 was dependent upon duration of antibody incubation. Control cells presented a slow cycling rate of FAT/CD36 and undetectable FAT/CD36 protein expression in plasma membrane fractions under basal conditions compared to OBT2D myotubes. Moreover decreasing the temperature at which the experiment was performed decreased cell surface FAT/CD36 in OBT2D myotubes consistent with a continuous cycling of FAT/CD36. As such, the increase in sarcolemmal FAT/CD36 observed in the skeletal muscle of type 2 diabetic patients may result from a continuous cycling of FAT/CD36 suggesting that defects in both, endocytosis and exocytosis may be expected. FAT/CD36 cycling was specific to myotubes and was not observed in reserve cells despite the presence of intracellular FAT/CD36. As such, we may suppose that FAT/CD36 cycling is controlled by a system specific to differentiated cells. Moreover, cell surface FAT/CD36 was observed in Control cells by incubating these cells with supernatants from OBT2D differentiated cells. This result implies that the appearance of cell surface FAT/CD36 is dependent upon a secreted factor or a combination of secreted factors or that a secreted factor inhibiting FAT/CD36 cycling is missing in differentiated OBT2D cell supernatant. Recent papers [27], [28] have shown by using proteomic approaches, that many proteins were secreted from human and mouse skeletal muscle cells and that these secreted proteins were modified in response to strength training or to TNF-alpha stimulation. Moreover, skeletal muscle secretome is dynamic during differentiation [29]. As such proteomic experiments should be performed in differentiated Control and OBT2D myotubes in order to identify secreted proteins which could be responsible for increased FAT/CD36 cycling during type 2 diabetes.

### The importance of FAT/CD36 cycling in determining lipid accumulation is different between OBT2D and Control myotubes

In adipocytes, FAT/CD36 expression appeared to be closely linked to preadipocyte differentiation [9]. Furthermore, FAT/CD36-mediated long-chain fatty acid uptake in adipocytes has been shown to require plasma membrane rafts [21]. In the present study, we showed that this is not the case in primary human muscle cells. As such, expression and localization of FAT/CD36 are differentially regulated during primary human muscle cell differentiation and murine preadipocyte differentiation. The fact that the proteins involved in fatty acids transport would differ in adipocytes and muscle tissue is not surprising given their different metabolic roles and physiological functions [30]. The precise localization of cell surface FAT/CD36 in relation to other known membrane proteins in primary human myotubes remains to be determined.

Fractionation experiments showed that only a minor part of FAT/CD36 was present in plasma membrane fractions implying that under basal condition, FAT/CD36 is mainly localized in intracellular compartments in OBT2D myotubes. Palmitate-induced lipid accumulation was observed in OBT2D differentiated cells with a concomitant cell surface FAT/CD36 localization. Moreover, the increase in palmitate-induced lipid accumulation observed in OBT2D myotubes was due to an increase in transporter-mediated lipid entrance (phloretin inhibition) mainly mediated by FAT/CD36 (SSO inhibition). Altogether these results show that cell surface localization of FAT/CD36 in fully differentiated OBT2D cells allows lipid accumulation. However, the more pronounced inhibition of lipid accumulation with phloretin (by 78%) compared to SSO treatment (by 47%) suggests that other lipid transporters may also be involved. This is consistent with the notion that membrane fatty acid binding protein contributes to fatty acid transport with FAT/CD36 [31], [32].

Acute AICAR or metformin treatment induces FAT/CD36 translocation in Control myotubes. This result was similar when using an adenovirus overexpressing a constitutively active form of AMPK, suggesting that FAT/CD36 translocation in response to AICAR or metformin treatment was due to AMPK activation. We hypothesized that similarly to OBT2D myotubes, increased FAT/CD36 translocation in Control myotubes would lead to increased lipid accumulation. However, inducing FAT/CD36 translocation in Control cells had no effect on lipid accumulation since AMPK activation in Control cells was able to significantly increase mitochondrial beta-oxidation. As such, increased translocation of FAT/CD36 in Control cells is not sufficient to induce lipid accumulation. We know from a previous work [15] that cells derived from obese type 2 diabetic patients display an impaired capacity to respond to metabolic stimuli at the level of mitochondrial activity, which could explain the difference in lipid content between OBT2D and Control cells even when control cells showed a similar cell surface FAT/CD36 content than OBT2D cells. Moreover, protein-mediated lipid transport was not the major mechanism responsible for palmitate-induced lipid accumulation in Control myotubes (less than 50% of inhibition by phloretin).

### Conclusion

This study is the first to show that increased lipid accumulation in OBT2D myotubes results from increased FAT/CD36 cycling. We propose that lipid content in Control myotubes may mostly be influenced by catabolic pathways (lipolysis, fatty acid oxidation) than anabolic pathways (uptake, esterification) in contrast to myotubes derived from obese type 2 diabetic patients.

## Materials and Methods

### Primary human satellite cell culture

Skeletal muscle biopsy of the vastus lateralis was performed according to the percutaneous Bergström technique after local anesthesia (xylocaine) [33], [34]. The experimental protocol was approved by “Le Comité de Protection de Personnes (CPP) «Sud Méditerranée IV»» (03/10/GESE, Montpellier, France). Informed and written consent was obtained from all subjects after explanation of the protocol. In the present study, biopsies were taken from four control subjects (control, age: 49.2±2.7 years, body mass index: 24.0±1.2 kg/m^2^) with no familial or personal history of diabetes and from five obese type 2 diabetic patients (OBT2D, age: 53.4±2.4 years, body mass index: 31.5±0.6 kg/m^2^). Clinical characteristics of the subjects were previously described [5]. The number of cell cultures used in this study is not high, however these cells were isolated from homogeneous populations of male subjects matched by age and physical activity [5]. Type 2 diabetic patients are obese since we have previously shown that cells derived from non obese type 2 diabetic patients did not show increased lipid accumulation compared to body mass index matched healthy subjects [5]. Cell culture of primary human satellite cells was performed as previously described [5], [12], [15], [35], cultures were maintained in a growth medium composed of (DMEM, 10% fetal bovine serum (FBS) and 1% Ultroser G) and when myoblasts reached confluence, medium was changed to differentiation medium (growth medium minus Ultroser G) and the differentiation process occurred until fusion and terminal differentiation into contractile myotubes (8 days). The experiments were performed on passages 2 to 4.

### Immunofluorescence

For intracellular staining, differentiated satellite cells were fixed with paraformaldehyde at 4% in PBS for 10 min, permeabilized with 0.5% triton in PBS for 2 min, saturated with 0.5% BSA in PBS for 10 min and then incubated with primary antibodies against troponin T diluted at 1/500 (Sigma, Saint-Quentin Fallavier, France), or polyclonal anti-CD36 diluted at 1/100 (H300, sc-9154, Tebu-BIO, Le Perray en Yvelines, France) or monoclonal anti-CD36 Alexa fluor 488 diluted at 1/100 (SM, Tebu-BIO, Le Perray en Yvelines, France). The antibody against FAT/CD36 (H300, sc-9154) was previously characterized on human skeletal muscle tissue and cells [5], [36], [37], [38], [39], [40]and the monoclonal anti-CD36 Alexa fluor 488 (SM, Tebu-BIO) was previously characterized in [5]. For troponin T staining, the secondary antibody was an anti-mouse coupled to Alexa 546 diluted at 1/1000 (GE Healthcare, Orsay, France) and for anti-CD36 (H300) an anti-rabbit coupled to Alexa 488 diluted at 1/1000 (GE Healthcare, Orsay, France).

FAT/CD36 has an extracellular loop allowing performing immunofluorescence in living cells by adding the antibody directly to the culture medium (FAT/CD36 staining in living cells can only be detected when the transporter is at the plasma membrane). We have used primary antibodies (SM) already coupled to fluorochrome (alexa 488 or PhytoErythrine) to avoid using a secondary antibody. Briefly, cells were washed with PBS and anti-CD36 Alexa Fluor 488 (SM) antibody was added at a dilution of 1/500 in PBS 2% BSA for 15 min, 1 h or 16 h either at 37°C or at room temperature (22°C) prior to paraformaldehyde fixation. For co-staining with GFP-expressing adenovirus, anti-CD36 PhytoErytrhine (CD36-PE, SM, Tebu-BIO) diluted at 1/500 was used instead of anti-CD36 Alexa fluor 488 (CD36-Alexa 488, SM, Tebu-BIO) (Fig. 3C).

For Figure 6C, medium from two independent differentiated Control (Control 1 and Control 2) cell cultures (day 7) was replaced by 1 ml of supernatant from two independent differentiated OBT2D (OBT2D 1 and OBT2D 2) cell cultures (Day 8) for 16 hours prior to staining on living cells with CD36 alexa 488.

### Virus infection

Adenovirus overexpressing a constitutively active truncated (40 kDa) form of AMPKα2 was previously described [19]. Infection of myotubes with either the empty adenovirus (GFP) or the adenovirus overexpressing the constitutively active form of AMPK alpha 2 (alpha 2) was performed on Day 6, 48 hours prior to the end of myotube differentiation.

### Fatty acid preparation and cells treatments

Stock solution (0.6 M) of palmitate was prepared. Palmitate was dissolved in chloroform. Samples were stored at −20°C until required. Fatty acid stocks were diluted to 0.6 mM in DMEM containing 10% FBS (differentiation medium) and added to the differentiated satellite cells. Control satellite cells were incubated in DMEM containing 10% FBS and 0,001% chloroform without palmitate as previously described [5], [12], [15]. Treatments performed on differentiated satellite cells are detailed in the figure legends and were realized in triplicate for each of the 9 independent cell cultures. The following reagents were purchased from Sigma (Saint Quentin Fallavier, France): L-Glutamine, DMEM, palmitate, phloretin, AMP-mimetic 5-aminoimidazole-4-carboxamide-1-β-D-ribofuranoside (AICAR), metformin and insulin. FBS was purchased from Hyclone (Brebieres, France) and Sulfo-N-succinimidyloleate (SSO) was a kind gift from W. Coumans (Maastricht, The Netherland).

### Oil red O staining

Oil red O staining was carried for one hour followed by 2 or 3 washes with distilled water. Lipid droplets were then visualized by light microscopy. In order to quantify lipid accumulation in myotubes, oil red O was extracted using isopropanol. The absorbance value was measured using a spectrophotometer set at 490 nm [41] and blanked to untreated cells. Staining and quantification of lipid accumulation in primary human satellite cells were previously described [5], [12], [15]. For Figure 2 D, conventional oil red O was modified according to the previously described protocol [42].

### Western Blots

Cellular extracts were quantified and lysed in Laemmli buffer. 30 µg of total proteins were transferred to nitro-cellulose membranes (Schleicher and Schuell, Bioscience, Dassel, Germany). Western blots were realized as previously described [8]. Following primary antibodies were used: anti-CD36 (H300, sc-9154) at 1/200; anti-caveolin 3 at 1/1000 and anti-BIP at 1/1000 (BD Biosciences, Le Pont de Claix, France); anti-troponin T at 1/500 and anti-α-tubulin at 1/1000 (Sigma Saint-Quentin Fallavier, France); The secondary antibodies were anti-rabbit and anti-mouse antibodies coupled to horseradish peroxydase diluted at 1/5000 (GE Healthcare, Orsay, France). Proteins were visualized using an enhanced luminescent reagent (Tebu-BIO, Le Perray en Yvelines, France), and exposed to autoradiograph film (GE Healthcare, Orsay, France). Expression of proteins was quantified by density analysis using ImageJ Launcher Software.

### Plasma membrane and post-plasma membrane fractions

One million of differentiated satellite cells derived from control subjects (Control) and type 2 diabetic patients (OBT2D) were used to prepare cell lysate, plasma membrane (Mb) and post-plasma membrane fractions (P) as previously described [43].

### Isolation of Detergent-Resistant Membranes (DRMs)

DRMs were prepared by detergent extraction of cellular proteins and centrifugation over discontinuous sucrose gradients essentially as described [44]. 4.5×10^6^ of differentiated satellite cells derived from control subjects (Control) and from obese type 2 diabetic patients (OBT2D) were used to prepare the DRMs. Cells were washed twice with ice-cold PBS and scraped into 667 µl of MBST buffer (25 mM MES, 150 mM NaCl, 1% Triton X-100, protease inhibitors mixture (Roche, Rosny-sous-bois, France) and homogenized with a loose fitting dunce homogenizer (20 strokes). The extract was adjusted to 40% sucrose by the addition of 667 µl of 80% sucrose in MBS lacking triton X-100 and placed in the bottom of an ultracentrifuge tube. A discontinuous sucrose gradient was formed by overlaying this solution with 1.33 ml of 38% sucrose and 1.33 ml of 5% sucrose (both in MBS). The tubes were centrifuged at 40,000 rpm in an SW60 Ti rotor for 16 h at 4°C, and 10 fractions (numbered 1 to 10) of 400 µl were collected manually from the top of the gradient, resuspended in Laemmli buffer and subjected to SDS-PAGE on 10% acrylamide gels followed by Western blot analyses.

### Palmitate oxidation

Control cells were cultured in 96-well plates and differentiated. Differentiated satellite cells were exposed or not to cold palmitate (0.6 mM) for the last 16 h of differentiation and then exposed to differentiation medium (DMEM+10% FBS) supplemented with 1% BSA, 50 µM palmitate and 9.5 µM (0,09 µCi) [1-^14^C]palmitate. Identical incubations were conducted on parallel plates that contained no cells. Palmitate oxidation rates were determined by measuring production of ^14^C-labeled acid-soluble metabolites (ASM), a measure of tricarboxylic acid cycle intermediates and acetyl esters. After incubation for 30 min at 37°C, reactions were terminated by aspiration of the media and addition of 100 µl of HClO_4_ at 5% for 15 min at room temperature. The ASM were assayed in supernatants of the acid precipitate. Radioactivity of ASM was determined by liquid scintillation counting by use of 4.5 ml of liquid scintillation cocktail (Optiphase ‘Hisafe’ 3, Perkin Elmer) in scintillation vials. For protein determination, identical incubations were conducted on parallel plates with the same number of cells.

### Statistical analysis

Statistical analyses were performed using Statview 5.0. Data are shown as mean ± SEM. Statistical analyses were performed using Student's t test for unpaired and paired comparison, or ANOVA with Fisher's PLSD post-hoc test for multiple comparisons. P<0.05 was considered to be significant.
